# Protective Effect of Ferulic Acid on Acetylcholinesterase and Amyloid Beta Peptide Plaque Formation in Alzheimer’s Disease: An In Vitro Study

**DOI:** 10.7759/cureus.54103

**Published:** 2024-02-13

**Authors:** Varsha Mugundhan, Abirami Arthanari, Parameswari R Parthasarathy

**Affiliations:** 1 Department of Forensic Odontology, Saveetha Dental College and Hospitals, Saveetha Institute of Medical and Technical Sciences, Saveetha University, Chennai, IND; 2 Department of Pharmacology, Saveetha Dental College and Hospitals, Saveetha Institute of Medical and Technical Sciences, Saveetha University, Chennai, IND

**Keywords:** neuroprotection, antioxidant, ferulic acid, amyloid, acetylcholinesterase, alzheimer’s disease

## Abstract

Aim

This study aims to comprehensively evaluate the effects of ferulic acid (FA) on acetylcholinesterase (AChE) enzyme activity and amyloid beta (Aβ) peptide plaque formation in an in vitro model of Alzheimer's disease (AD).

Background

AD is a progressive neurological condition marked by disrupted cholinergic signaling, accumulation of Aβ peptide, and tau protein hyperphosphorylation. Currently, no direct anti-Alzheimer drug that effectively prevents the cognitive decline from AD has been reported. To combat this, a multi-target drug addressing several molecular aspects would be ideal for AD. Natural compounds are preferred over synthetic drugs due to their accessibility, cost-efficiency, and lower toxicity The proven association between polyphenol consumption and the prevention of AD has led to the investigation of the effect of FA, a polyphenolic compound, on acetylcholinesterase enzyme activity and Aβ peptide formation, the key targets of AD.

Materials and method

The free radical scavenging ability of FA was assessed by xanthine oxidase inhibitory activity. Furthermore, FA was also evaluated for its inhibitory activity against AChE enzyme and amyloid beta peptide formation to evaluate the neuroprotective potential of FA.

Results

The results showed that FA has the potential to be an AChE inhibitor, thus helping in blocking the activity of AChE and also reducing the incidence of amyloid beta plaque formation. Furthermore, the compound also exhibited a significant antioxidant property which was demonstrated by the xanthine oxidase enzyme inhibitory effect.

Conclusion

From the observed results, FA has significant antioxidant and neuroprotective effects which are compared with those of their respective standards. More research is required to determine the efficacy and safety of this compound as a treatment for neurodegenerative diseases like AD because the precise mechanism and degree of its AChE inhibitory effects in the brain are still elusive. A potent, selective, and effective drug is desperately needed to treat patients with AD and those at risk of developing the disease.

## Introduction

Alzheimer's disease (AD) is a disorder that causes cell degeneration in the brain and is the leading cause of dementia, which is characterized by a decline in thinking and independence in daily activities. AD is thought to be a multifactorial disease, with two main hypotheses proposed as causes: the cholinergic and amyloid hypotheses [1]. Presently, it impacts more than 40 million individuals globally, constituting about 70% of all dementia cases. According to recent epidemiological data, by 2050, more than three-quarters of AD and related dementias (ADRD) cases are projected to arise in countries with lower and moderate income levels. A very recent report has demonstrated that more than 8.8 million individuals (older than 60 years) in India have been diagnosed with dementia [2]. The symptoms of AD involve a gradual decline in cognitive functions concomitant with permanent loss of neurons. In Alzheimer's, senile plaques develop due to the accumulation of amyloid beta protein (Aβ). Aβ42 variants, ending at Ala42, tend to form these plaques more than Aβ40 (Val40) in affected brains. This starts with amyloid precursor protein converting into Aβ42, leading to abnormal clusters and fibrils. These trigger synaptic issues, tau protein accumulation, nervous system inflammation, and oxidative stress, culminating in neuronal death and cognitive decline progression [3]. The cholinergic neurotransmission has been proposed as the primary and earliest impacted molecular pathway delineating the pathophysiology of AD [4]. Only a few drugs such as rivastigmine, galantamine, and donepezil have been approved for the prevention and treatment of AD. However, the treatment approaches formulated to address AD-related neuronal deterioration have been partially successful because they rely on single-target medications that are incapable of attenuating the enduring decline in brain functions [5]. Current investigations focus on discovering novel compounds with the dual capacity to address AD by targeting cholinergic neurotransmission similar to acetylcholinesterase inhibitors (AChE-Is). These compounds should also possess the ability to inhibit the formation and deposition of Aβ (amyloid-beta) while concurrently reducing oxidative stress [4]. Therefore, considerable attention is currently directed toward an AD treatment strategy involving multiple targets, intervening across various pathways concurrently, and lifestyle interventions aimed at mitigating modifiable risk factors.

Accumulating evidence suggests that a diet enriched with vitamins, polyunsaturated fatty acids, polyphenols, and flavonoids along with dietary restrictions may lower the risk of AD [5]. In recent times, the Mediterranean diet has garnered attention for its potential to reduce not only the risk of neurodegenerative disorders but also heart diseases and various types of cancer. This dietary approach has been demonstrated to be abundant in plant-based elements and rich in polyphenolic phytochemicals which is attributed to its promising preventive aspects [6]. Polyphenols possess antioxidant properties, and various animal studies have explored their link to amyloid accumulation and reducing oxidative stress in individuals with AD [7]. Hartman et al. discovered that supplementing mice with pomegranates, high in polyphenols, lowered AD risk by 50% compared to a control group [8]. Similarly, Kim et al. observed that resveratrol from grapes lessened hippocampal neurodegeneration and improved learning in a transgenic mouse model [9]. In view of the given background, in the present study ferulic acid (FA), a well-known polyphenolic compound, has been investigated for neuroprotective properties.

FA, a type of phenolic acid, is commonly present in natural plants. It is frequently bound with lignin and polysaccharides to create plant cell walls and is seldom found in a free state. FA, chemically identified as 4-hydroxy-3-methoxy cinnamic acid (C10H10O4, with a molecular weight of 194), is a derivative of cinnamic acid (3-phenyl-2-acrylic acid) exhibiting both cis and trans structural forms [10]. Pharmacological investigations indicate that FA demonstrates antioxidant, free radical-scavenging, anti-thrombotic, lipid-lowering, antibacterial, antiviral, anti-mutagenic, and anticancer properties [11]. Additionally, studies report that FA exhibits anti-inflammatory and antioxidant properties in cerebral ischemic rat models [12]. FA has been demonstrated for its significant neuroprotective properties against various experimental models viz., brain injury, spinal ischemia, and pathology resembling Alzheimer's disease [13,14]. Furthermore, FA mitigated neuroinflammatory responses and ameliorated behavioral impairments in a mouse model of Parkinson's disease [15]. Furthermore, FA demonstrates inhibitory effects on the inflammatory reactions triggered by lipopolysaccharides (LPS) in microglia. Studies indicate its ability to modulate β-secretase activity, ameliorating AD-like pathology in transgenic mice models. FA has also been observed to inhibit acetylcholinesterase (AChE) activity and restore mitochondrial membrane potential [16]. Taken together, it might be suggested that FA has the potential to reduce the susceptibility to AD development and a significant role in disease management. Furthermore, prior to initiating clinical trials for new drugs, it is essential to conduct in vitro studies focusing on target enzymes/receptor profiling [17]. Therefore, the present study aimed to investigate the effect of FA on the in vitro enzymatic inhibitory activity of AChE and the formation of Aβ plaques.

## Materials and methods

Reagents and chemicals

Xanthine, acetylthiocholine iodide, and acetylcholine enzyme (0.3Units/ml) were procured from Sigma-Aldrich, USA. FA and quercetin were purchased from TCI Chemicals, India. Donepezil hydrochloride was purchased commercially in tablet form from a local pharmacy. All other chemicals, reagents, and solvents were of analytical grade and purchased from SRL chemicals in India.

Dose fixation for enzymatic inhibitory activity

FA was assessed for its antioxidant and neuroprotective properties through in vitro enzymatic inhibition tests targeting xanthine oxidase (XO) and acetylcholinesterase. Additionally, its ability to inhibit the formation of amyloid fibrils was demonstrated. Testing involved varying concentrations of FA (10, 20, 40, 80, 160 & 320 µM). The chosen FA concentrations were based on previous studies showcasing its efficacy in vitro for antioxidant, anti-inflammatory, and neuroprotective effects. The reference drug, Donepezil hydrochloride, was obtained as a 5mg tablet from the pharmacy. Preclinical rodent studies demonstrated significant cognitive enhancement with Donepezil at 3mg/kg b.wt., prompting the selection of an approximately ten-fold lower dose (0.32mg/ml or 320μg/ml) as the maximum concentration, with other concentrations determined through a serial dilution method. Various concentrations of FA, quercetin, and donepezil were chosen to assess the inhibitory response of the compounds against the enzymes in a dose-dependent manner. Experiments were conducted in 96-well microplates, and the absorbances were recorded utilizing a microplate reader (Biotek Synergy H4, Biotek Instruments, Winooski, USA).

XO inhibitory activity

The XO inhibitory activity was assayed spectrophotometrically, based on the procedure reported by Hudaib et al. 2011 [18]. The substrate and the enzyme solutions were freshly prepared. The assay mixture, consisting of 50 $\mu$L of different concentrations FA (10-320 $\mu$M), different concentrations of Quercetin (10-320$\mu$M, 35 $\mu$L of 0.1mM phosphate buffer (pH=7.5) and 30 $\mu$L of enzyme solution (0.01units/ml of XO in 0.1mM phosphate buffer, pH=7.5), was prepared immediately before use. After 30 minutes of incubation at 25°C, the reaction was initiated by the addition of 60 $\mu$L of substrate solution (150 mM of Xanthine in 0.1 mM Phosphate buffer). The absorption at 295 nm, indicating the formation of uric acid at 25°C, was monitored and the initial rate was calculated. A blank was prepared in the same manner. One unit of XO was defined as the amount of enzyme required to produce 1 mmol of uric acid/minute at 25°C. XO inhibitory activity is expressed as the percentage inhibition of XO in the above system, calculated as (1-B/A) × 100, where A and B are the activities of the enzyme without and with different concentrations of FA and quercetin. IC50 values were calculated from the mean values of data from three determinations. Quercetin was used as a reference standard.

In vitro acetylcholinesterase (AChE) inhibition assay

The FA and standard donepezil hydrochloride were examined for their AChE inhibitory activities at different concentrations of 10-320 $\mu$M and 10-320 $\mu$g/ml respectively [19]. 200 $\mu$l of the different concentrations of FA and standard donepezil hydrochloride were prepared using 0.05 M tris base. Briefly, in this method, 200$\mu$l of acetylthiocholine iodide (15mM), 1000 $\mu$l of DTNB (3mM), and 200 $\mu$l of different concentrations of FA and donepezil were mixed and incubated for 15 minutes at 30°C. The mixture was then observed spectrophotometrically at 412 nm for 10 times 13s. Then, 200 $\mu$l of AChE solution (0.3 U/ml) was added to the original mixture to start the reaction, and then the absorbance was determined. The control contained all components except the tested extract. The percentage of AChE inhibitory activity (% IA) was calculated using the following equation:$\mu$

IA (%) = (Activity of Control - Activity of Test)/ Activity of Control x 100

Assessment of amyloid beta (Aβ (1-42)) concentration preparation of Aβ solution

Aβ solution was prepared according to the method of Miyazaki et al., 2019 [20]. Briefly, synthetic β-amyloid peptide 1, 42 (Aβ1, 42) (PP69, Sigma Merck, USA) dissolved in 0.1% ammonium solution at a final concentration of 250 $\mu$M and sonicated in cold water for a total of 5 min (1 min × 5 times) to avoid the pre-association. To prepare the Aβ solution, aliquots of Aβ were diluted to the 25 $\mu$M in 50 mM phosphate buffer (pH 7.5) and 100 mM NaCl.

Thioflavin T (Tht) fluorescence assay

Miyazaki et al., (2019) described the thioflavin T fluorescence assay also was completed [20]. FA (10-320 $\mu$M) and donepezil (10-320 $\mu$g/ml) were combined in a solution (8 L), and the resulting mixture was then added to 1.6 mL of ThT solution, which contained 5 M ThT and 50 M NaOH-glycine-buffer (pH 8.5). ThT fluorescence tests were used to monitor the fibrillogenesis rate while the models were incubated at 37°C. Using a Biotek Synergy H4 hybrid multimode reader, the samples' ThT fluorescence levels were assessed. 446 nm and 490 nm were the excitation and emission wavelengths, respectively.

Statistical analysis

GraphPad Prism (version 7, GraphPad Software, San Diego, CA) was used to statistically analyze the data. The data were presented as Mean±Standard Error Mean, and the values for IC50 were derived from the plots of the linear regression. To evaluate mean differences at the p<0.001 level of significance, two-way ANOVA was utilized. The Holm-Sidak Test was used to compare the means to comparison groups of standards.

## Results

XO activity

**Figure 1:**
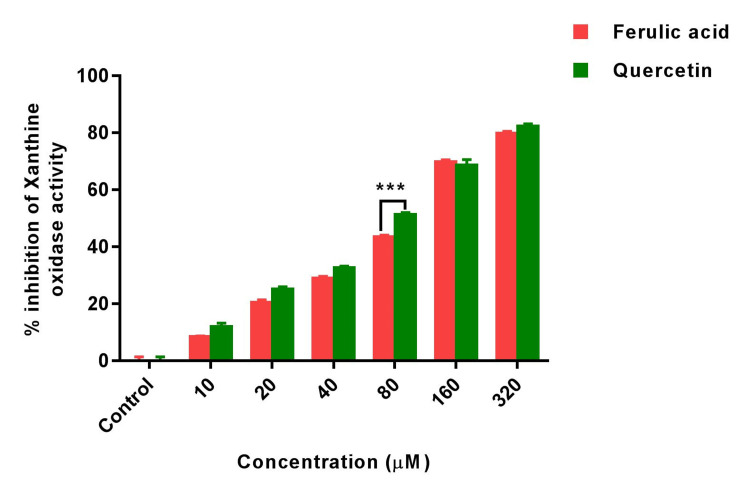
Bar graph shows the xanthine oxidase inhibitory activity of ferulic acid and standard quercetin. Statistical analysis was performed using two ANOVA followed by Holm-Sidak’s test. Results are expressed as Mean±SEM (n=3). (***) of FA significantly different from Standard quercetin at p<0.001.

XO, known for its role in generating hydrogen peroxide and inducing oxidative stress, was assessed in order to decipher the antioxidant potential of FA. We assessed the inhibitory effect of FA across a range of concentrations (10-320 $\mu$M) on XO activity and compared its efficacy to the standard quercetin. Our findings revealed that FA, at concentrations ranging from 10 $\mu$M to 320 $\mu$M, demonstrated substantial inhibition of XO, with inhibitory rates ranging from 5.6±0.62% to 77±0.24% compared to the quercetin standard. Quercetin, as a standard, displayed an inhibitory effect on XO activity within the range of 7±0.66% to 79.8±0.54%. Notably, at 80 $\mu$M concentration, FA exhibited significant differences (p<0.001) compared to quercetin, indicating variability at this specific concentration, while other concentrations exhibited comparable inhibition levels to the quercetin standard (Figure 1).

AChE inhibitory activity

**Figure 2:**
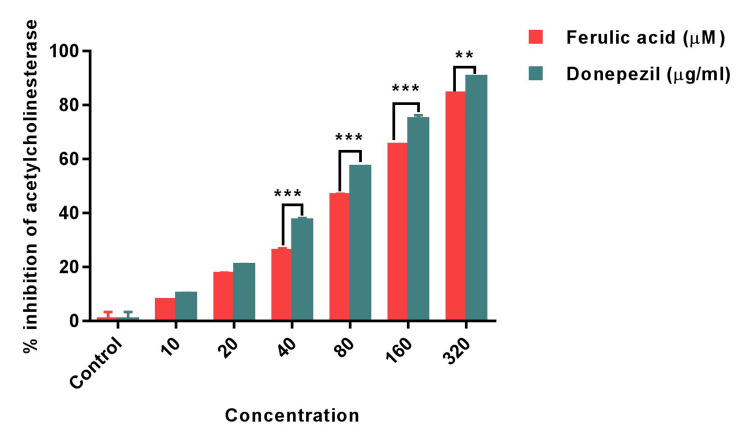
Bar graph shows the acetylcholinesterase inhibitory activity of Ferulic acid and standard Donepezil. Statistical analysis was performed using two ANOVA followed by Holm-Sidak’s test. Results are expressed as Mean±SEM (n=3). (***) of FA significantly different from Standard Donepezil at p<0.001. (**) of FA significantly different from Standard Donepezil at p<0.01.

The primary focus of potential AD treatments revolves around AChE, considered a promising therapeutic target. Among various drug options, AChE inhibitors are leading candidates. In this study, FA's AChE inhibitory activity was assessed across different concentrations. The findings depicted significant AChE inhibition by FA, compared to the standard cholinesterase inhibitor, Donepezil. FA exhibited inhibitory activity ranging from 2.4±0.12% to 81.8±0.82% at concentrations between 10 $\mu$M and 320 $\mu$M, whereas Donepezil, the standard Alzheimer's drug, demonstrated 86±0.34% inhibition against AChE at 320 $\mu$M. Notably, concentrations of 10 $\mu$M and 20 $\mu$M did not exhibit substantial variation in AChE activity between FA and Donepezil. However, concentrations ranging from 80 $\mu$M to 320 $\mu$M showed a significant (p<0.001) difference. At 320 $\mu$M, FA showcased an inhibitory effect above 80%, suggesting a potent anti-cholinergic impact (Figure 2). 

Inhibition of amyloid beta peptide aggregation

**Figure 3:**
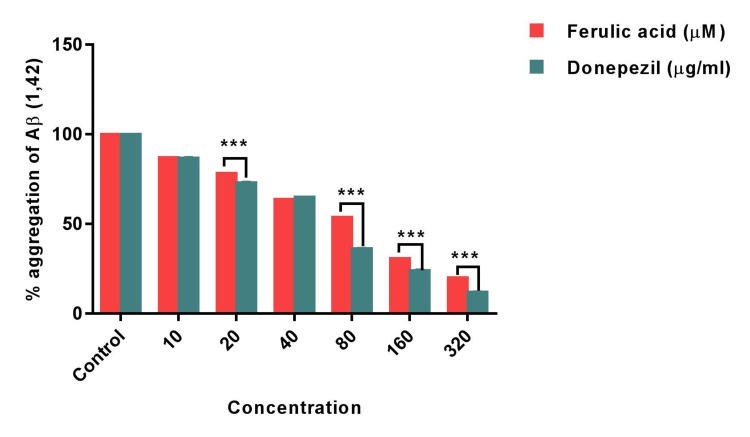
Bar graph shows the percentage aggregation of Ab (1, 42) exhibited by ferulic acid and standard donepezil. Statistical analysis was performed using two-way ANOVA followed by Holm-Sidak’s test. Results are expressed as Mean±SEM (n=3). (***) of FA significantly different from standard donepezil at p<0.001. (**) of FA significantly different from standard donepezil at p<0.01.

Furthermore, FA was evaluated for its potential against the accumulation of amyloid β peptide which is one of the hallmarks of AD. The study findings showed that aggregation of amyloid β peptide was significantly inhibited by FA which was also compared with standard donepezil (Figure 3).

**Figure 4:**
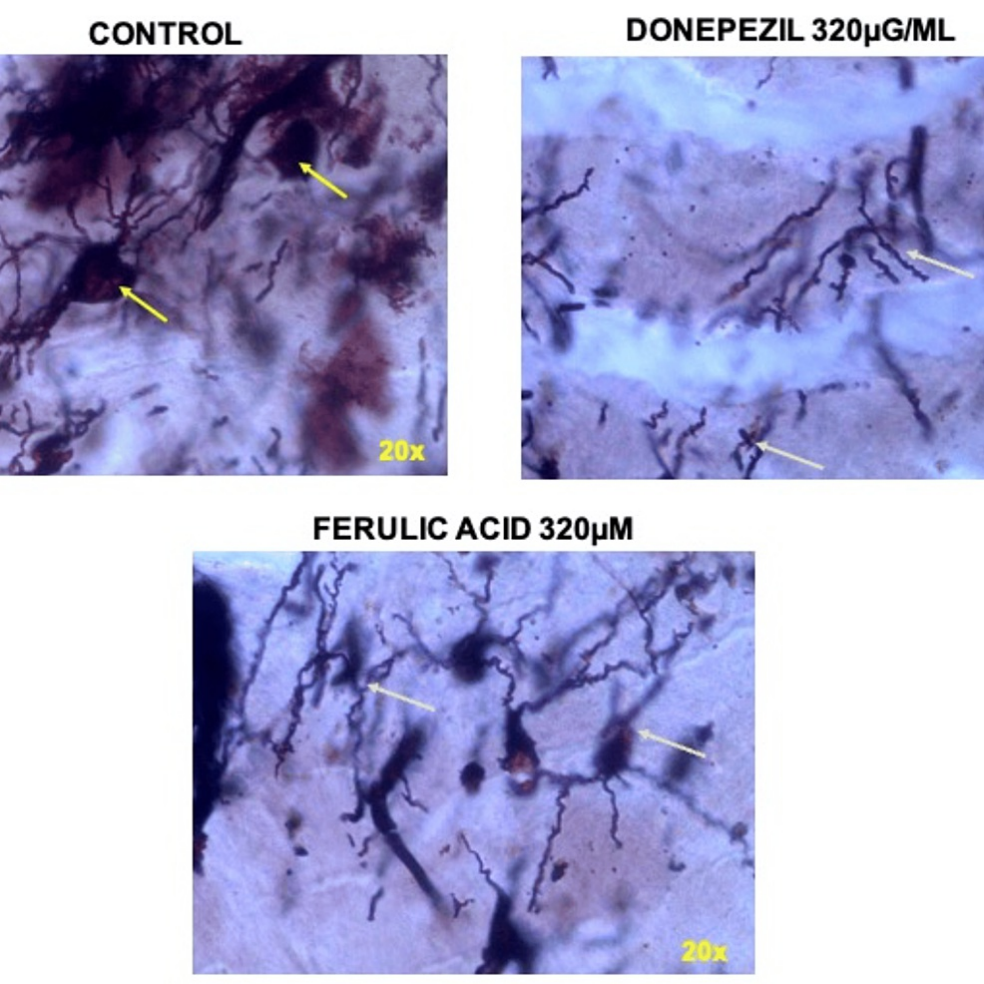
Representative microscopic images showing amyloid fibril formation (tangles like) in control group (yellow arrowheads). Donepezil (320μg/ml) and ferulic acid (320μM) exhibited reduction in the amyloid fibril formation (green arrowheads).

Although the donepezil has exhibited significant inhibition, the effect of FA was comparable with the standard’s effect with maximum inhibition of 80% at highest concentration of 320 $\mu$Μ. The 10 $\mu$M concentration of FA and donepezil exhibited similar percentage (84.2±0.88% and 85.4±0.26% respectively) aggregation of Aβ (1, 42) peptide. However, the 20 $\mu$Μ FA and concentrations of FA between 80 $\mu$Μ - 320 $\mu$Μ has demonstrated significant difference (p<0.001) in the percentage of Aβ (1, 42) peptide aggregation. Intriguingly, the concentration 40 $\mu$Μ of FA showed a mild higher percentage inhibition in the Aβ (1, 42) peptide aggregation (Figure 4).

## Discussion

In this study, the antioxidative and neuroprotective properties of FA were examined. The findings demonstrated significant suppression of XO activity by FA, indicating its capacity to scavenge free radicals. Additionally, FA was assessed for its ability to inhibit acetylcholinesterase activity and amyloid beta peptide formation. The results revealed a marked reduction in acetylcholinesterase activity by FA, consequently mitigating the production of amyloid beta peptide (Aβ 1, 42). These outcomes suggest a potential preventive effect of FA against AD.

Literature has shown that the brain of AD patients exhibited significant damage related to oxidative stress, such as protein oxidation, lipid peroxidation, and DNA damage. This indicates an imbalance between the production of free radicals and the brain's antioxidant activity. The reduced antioxidant activity in the brain leads to increased amyloid beta production, exacerbating damage to proteins and lipids. The primary origin of oxidative stress in the Alzheimer's brain stems from free radicals produced by mitochondria and redox-active metals. This elevation in oxidative stress further contributes to membrane dysfunction, alterations in the cytoskeleton, and neuronal cell death [21]. XO, a major source of reactive oxygen species (ROS) facilitates the conversion of oxygen into a superoxide anion, leading to hydrogen peroxide formation. Excessive hydrogen peroxide can induce cytotoxic effects resulting in heightened oxidative stress within cells [22]. Polyphenol’s therapeutic effectiveness is linked to its strong antioxidant properties and natural radical scavenging ability. Studies have already shown that polyphenolic compounds like nobiletin, quercetin, apigenin, and curcumin have markedly attenuated oxidative stress by scavenging the free radicals with concomitant elevation in the antioxidant levels [23]. FA, known for its antioxidative properties, has undergone extensive investigation due to its capacity to scavenge ROS and enhance various antioxidant enzyme activities, including SOD and catalase, under conditions of induced oxidative stress. Prior research has evidenced the restoration of SOD activity in diabetic rats following FA treatment [24]. Similarly, in hypoxia-induced PC12 cells, FA has demonstrated the restoration of SOD activity, mitigated lipid peroxidation, and exhibited free radical scavenging capabilities [25]. Studies have also delineated the intricate antioxidant mechanisms of FA, primarily involving the inhibition of ROS or nitrogen generation and the neutralization of free radicals [26]. The current study's findings align with these previously established observations, revealing significant inhibition of xanthine oxidase, a major ROS source by FA treatment, suggesting FA's potential to mitigate oxidative stress and potentially impede the progression of AD thereby providing neuroprotection.

The cholinergic system has been proposed as an initial and extensively investigated molecular mechanism in the pathophysiology of AD. It represents the primary degenerative process responsible for the impairment of cholinergic neurons in the brain, leading to cognitive decline. The interplay between neurofibrillary alterations and β-amyloid pathology profoundly influences cholinergic receptors, hastening AD progression [26]. Curcumin has been shown to interfere in the synthesis of amyloid beta peptide thereby preventing the accumulation of Aβ. Similarly, quercetin has also been shown to interfere with the synthesis of Aβ by inhibiting the β-Secretase activity [23]. In line with these earlier findings on the neuroprotective potential of other polyphenolic compounds, substantial evidence has highlighted FA's neuroprotective (anti-amyloid) properties and its capacity to inhibit AChE [27,28]. A composite compound of FA and styryl benzene displayed inhibition of Aβ fibril formation, indicating potential interaction with non-fibrous or monomer-like Aβ structures [29]. Additionally, FA, when combined with tacrine, demonstrated inhibitory effects on acetylcholinesterase and butyrylcholinesterase. Notably, tacrine-6-ferulic acid exhibited heightened choline acetyltransferase activity and acetylcholinesterase inhibition in an AD mouse model, potentially impacting Aβ self-aggregation and enhancing memory in mice [28]. Further investigations revealed FA's reduction in cerebral amyloid accumulation and its decrease in β-secretase activity in PSAPP mice, suggesting a mechanism involving BACE1 destabilization [27]. In summary, FA showcases anti-amyloidogenic effects by impeding AChE function and destabilizing the β-secretase enzyme, thus improving behavioral impairments associated with AD. The current study's outcomes align with prior findings, indicating substantial acetylcholinesterase inhibition and suppression of amyloid beta peptide formation, affirming FA's potent neuroprotective role against neurodegeneration and AD.

Limitations of the study

It is important to note that while these assays offer significant insights into FA's potential therapeutic effects, further studies should address certain aspects for a more comprehensive understanding. The precise mechanism by which FA exerts its AChE inhibitory effects on the brain warrants deeper exploration. Additionally, studies on the compound's bioavailability, pharmacokinetics, and long-term safety profile are essential to ascertain its suitability as a viable treatment option for AD.

## Conclusions

In conclusion, FA emerges as a promising compound in the realm of AD research. Its multifaceted actions, including antioxidant and anti-amyloidogenic properties and inhibition of key enzymes like AChE, showcase its potential as a neuroprotective agent against neurodegeneration and AD-related pathology. The observed effects on cholinergic function and amyloid burden reduction, along with a potent antioxidant effect, underscore FA's therapeutic promise. However, while these findings are encouraging, further comprehensive studies and clinical investigations are imperative to ascertain FA's efficacy, safety, and suitability for potential therapeutic interventions in AD.
